# Concizumab in patients with hemophilia A or B without inhibitors: 56-week cutoff results of the phase 3 explorer8 study

**DOI:** 10.1182/bloodadvances.2026019931

**Published:** 2026-06-26

**Authors:** Guy Young, Pantep Angchaisuksiri, Shashikant Apte, Renée Brown Frandsen, Anthony K. C. Chan, Pratima Chowdary, Hermann Eichler, Chuhl Joo Lyu, Maria Fernanda Martinez Garcia, Tadashi Matsushita, Sonata Šaulytė Trakymienė, Hyuen Tran, Alice Trinchero, Jerzy Windyga, Jan Astermark

**Affiliations:** 1 Hemostasis and Thrombosis Center, Cancer and Blood Disease Institute, Children’s Hospital Los Angeles, Keck School of Medicine, University of Southern California, Los Angeles, CA; 2 Division of Hematology, Department of Medicine, Ramathibodi Hospital, Mahidol University, Bangkok, Thailand; 3 Department of Haematology, Sahyadri Specialty Hospitals, Pune, India; 4 Novo Nordisk A/S, Søborg, Denmark; 5 Department of Pediatrics, McMaster University, Hamilton, ON, Canada; 6 Katharine Dormandy Haemophilia and Thrombosis Centre, Royal Free London NHS Foundation Trust, London, United Kingdom; 7 Institute of Clinical Hemostaseology and Transfusion Medicine, Saarland University and University Hospital, Homburg, Germany; 8 Department of Paediatric Haematology-Oncology, Yonsei Cancer Center, Severance Hospital, Yonsei University College of Medicine, Seoul, Republic of Korea; 9 Department of Transfusion Medicine, Nagoya University Hospital, Nagoya, Japan; 10 Clinic of Children’s Diseases, Faculty of Medicine, Vilnius University Hospital Santaros Klinikos, Vilnius University, Vilnius, Lithuania; 11 Ronald Sawyers Haemophilia Centre, The Alfred Hospital, Melbourne, VIC, Australia; 12 Australian Centre for Blood Diseases, Monash University, Melbourne, VIC, Australia; 13 Clinic for Medical Oncology and Haematology, University Hospital Zurich, Zurich, Switzerland; 14 Department of Hemostasis Disorders and Internal Medicine, Laboratory of Hemostasis and Metabolic Diseases, Institute of Hematology and Transfusion Medicine, Warsaw, Poland; 15 Department of Translational Medicine, Lund University, Skåne University Hospital, Malmö, Sweden; 16 Department of Hematology, Oncology and Radiation Physics, Skåne University Hospital, Malmö, Sweden

## Abstract

Concizumab is a novel nonfactor replacement therapy for once-daily subcutaneous prophylactic treatment of hemophilia A/B (HA/HB) with and without inhibitors. Concizumab was superior to on-demand treatment in patients with HA/HB without inhibitors in the prospective, multicenter, open-label phase 3 explorer8 study. Here, longer-term efficacy and safety results from the start of the study up to the 56-week cutoff are presented. Males aged ≥12 years with HA/HB were randomized 1:2 to no prophylaxis (group 1) or concizumab (group 2) or allocated to concizumab (groups 3 and 4). Assessments at the 56-week cutoff included efficacy, pharmacokinetics/pharmacodynamics, and safety. The 56-week cutoff was defined as when all patients in groups 2 to 4 had completed the visit at 56 weeks or permanently discontinued treatment. Of 148 patients in the full analysis set, 21 were randomized to no prophylaxis (group 1: HA, n = 9; HB, n = 12), 42 to concizumab (group 2: HA, n = 18; HB, n = 24), and 85 to the nonrandomized concizumab groups (groups 3 and 4: HA, n = 55; HB, n = 30). After ≥24 weeks of treatment, 17 patients in group 1 switched to concizumab. Low median annualized bleeding rates for treated spontaneous and traumatic bleeding episodes were maintained at the 56-week cutoff in patients receiving concizumab (HA, 1.7 [interquartile range (IQR), 0.0-4.5]; HB, 2.8 [IQR, 0.0-6.4]), consistent with 32-week cutoff results. Concizumab plasma concentration remained stable, with no new safety concerns. Concizumab showed longer-term efficacy in patients with HA/HB at the 56-week cutoff and was considered safe and well tolerated. This trial was registered at www.clinicaltrials.gov as #NCT04082429.

## Key Points

• Longer-term (≥1 year) efficacy and safety data for concizumab were consistent with the 32-week cutoff results in the explorer8 study.
• Low bleeding rates were maintained on concizumab, with no new safety concerns in patients with HA/HB without inhibitors.

## Visual Abstract

**Figure:**
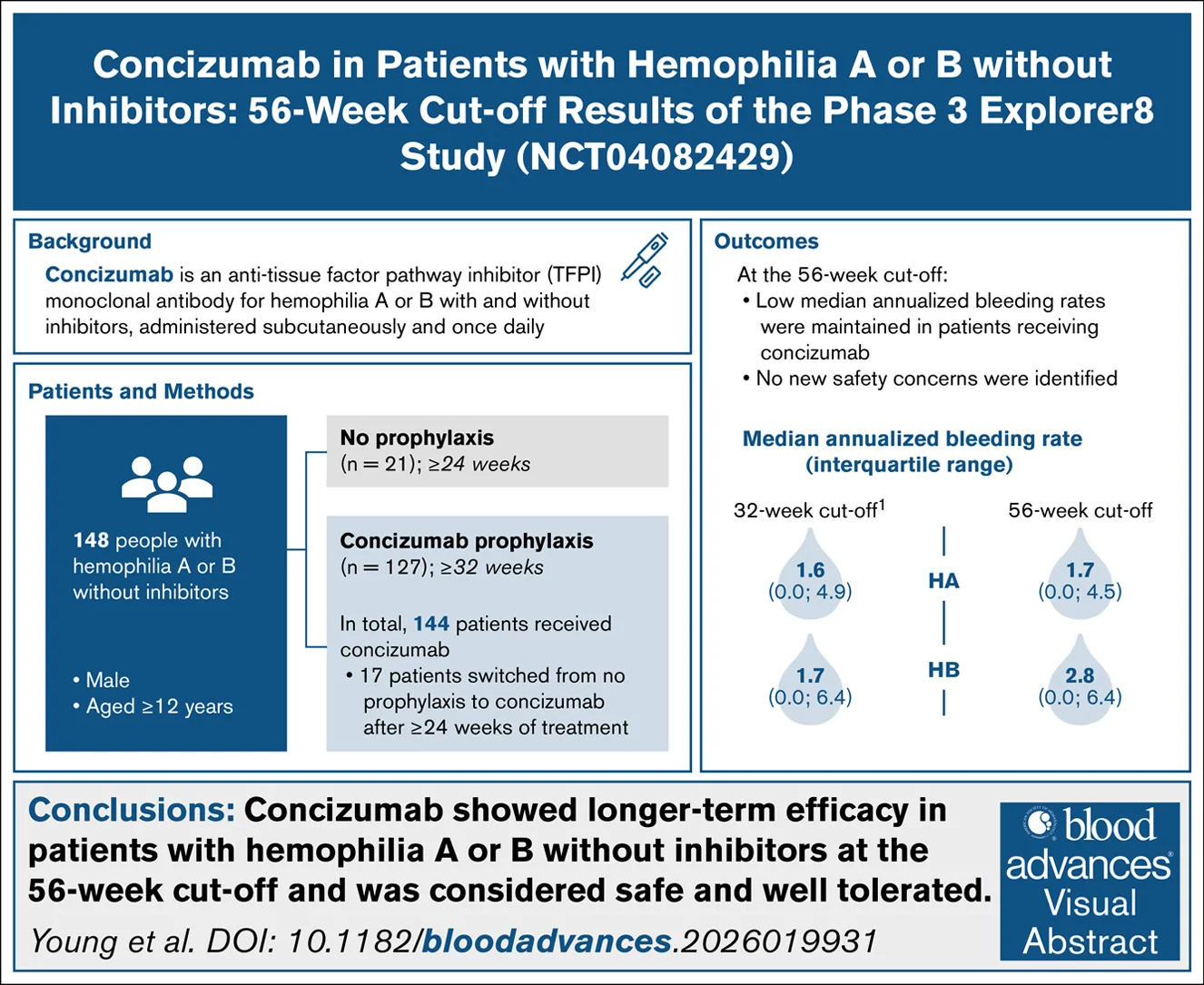

## Introduction

Concizumab is a novel nonfactor replacement therapy for hemophilia A (HA) and hemophilia B (HB). Patients with HA/HB have an increased tendency toward potentially life-threatening bleeds compared with healthy individuals, caused by a deficiency of either coagulation factor VIII (FVIII; HA) or factor IX (FIX; HB).^1^ Current standard of care typically involves IV prophylactic treatment that replaces the deficient coagulation factor or subcutaneous nonfactor replacement therapies.^2^ The development of inhibitors (neutralizing antibodies) to factor replacement products in patients decreases the effectiveness of factor replacement therapy, leading to treatment complexities and increasing burden.^3^ Despite a range of existing treatments and the availability of nonfactor therapy in HA, significant unmet needs persist, particularly for patients with HB who have IV access issues and limited licensed subcutaneous nonfactor treatment options.^4^

Concizumab is a humanized anti–tissue factor pathway inhibitor (TFPI) monoclonal antibody that acts by preventing the inhibition of coagulation by TFPI, thereby restoring thrombin generation. Concizumab has established its efficacy and safety, regardless of hemophilia type and inhibitor status, during phase 3 trials.^5^^,^^6^ In patients with HA/HB with inhibitors (explorer7 [ClinicalTrials.gov identifier: NCT04083781]) and without inhibitors (explorer8 [ClinicalTrials.gov identifier: NCT04082429]), daily subcutaneous concizumab administration significantly reduced the number of bleeding episodes vs on-demand treatment with bypassing agents or clotting factor concentrate.^5^^,^^6^ Further, concizumab plasma concentration remained stable over time, and treatment was considered safe and well tolerated.^5^^,^^6^

Here, results from longer-term concizumab use in the phase 3 explorer8 study at the 56-week cutoff in patients with HA/HB without inhibitors are presented, extending the confirmatory analysis results reported at the 32-week cutoff.^5^

## Methods

### Study design

The explorer8 study design has been previously described.^5^ Briefly, explorer8 was a prospective, multicenter, open-label phase 3a clinical study evaluating the efficacy and safety of daily concizumab prophylaxis in males aged ≥12 years (>25 kg) with severe HA (FVIII <1%) or moderate/severe HB (FIX ≤2%) without inhibitors.

The study included 4 groups (supplemental Figure 1); patients receiving on-demand treatment with no previous exposure to concizumab were randomized 1:2 to group 1 to continue on-demand treatment (no prophylaxis group) or to group 2 to receive concizumab prophylaxis (concizumab prophylaxis group). Group 1 patients received concizumab prophylaxis and continued into the extension part of the study once they had completed ≥24 weeks of on-demand treatment.

Patients who transferred from the phase 2 noninhibitor study (explorer5 [ClinicalTrials.gov identifier: NCT03196297]) or from the noninterventional study (explorer6 observational study [ClinicalTrials.gov identifier: NCT03741881]) and other patients detailed in supplemental Figure 1 were allocated to nonrandomized groups 3 and 4 and received concizumab prophylaxis. Patients allocated to group 4 who entered explorer8 from the noninterventional explorer6 study were included in an intrapatient comparison of concizumab to previous stable prophylaxis (defined in explorer6 as ≥24 weeks of local standard-of-care factor prophylaxis).

The 56-week cutoff was defined as when all patients in groups 2 to 4 had completed the visit at 56 weeks or permanently discontinued treatment. The data used for the analyses were collected from the start of the study up to the 56-week cutoff. For patients in group 1, data collected up to this time point were evaluated as part of the reporting of results at the 56-week cutoff.

Concizumab treatment was paused from March 2020 to August 2020 because of nonfatal thromboembolic events in 2 patients in explorer8 and 1 patient in explorer7.^5^^,^^6^ All 3 patients were found to have different thromboembolic risk factors at baseline. After thorough investigation of all data, treatment was restarted with the implementation of risk mitigation measures that included a revised dosing regimen and bleed management guidance. Patients randomized or allocated to concizumab prophylaxis groups received a 1.0 mg/kg concizumab loading dose on day 1, followed by daily doses of 0.20 mg/kg starting from day 2 onward. Based on concizumab plasma concentration measured after week 4 (concizumab plasma concentration range for maintenance dose adjustment, 200-4000 ng/mL), the concizumab maintenance dose was adjusted to 0.15 mg/kg if >4000 ng/mL or to 0.25 mg/kg if <200 ng/mL during weeks 5 to 8 or maintained at 0.20 mg/kg if 200 to 4000 ng/mL, therefore allowing for a 1-time dose adjustment guided by concizumab plasma concentration (supplemental Figure 1).

The study was approved by independent ethics committees/institutional review boards and conducted in accordance with the Declaration of Helsinki and the International Council for Harmonisation Good Clinical Practice.

### Assessments

Assessments at the 56-week cutoff included efficacy, pharmacokinetics/pharmacodynamics (PK/PD), and safety measures until the 56-week cutoff, all descriptively presented herein.

Efficacy was assessed as the number of treated spontaneous and traumatic bleeding episodes from the start of the new concizumab dosing regimen after the treatment restart until the 56-week cutoff for patients in groups 1 to 4,^7^ including the subset of patients in group 4 who were part of the intrapatient comparison of concizumab prophylaxis to previous prophylaxis.^8^ Further efficacy exploratory analyses included target joint status at baseline, the location of target joints, and the number of treated spontaneous and traumatic joint and target joint bleeding episodes.^9^ Target joints were defined as ≥3 spontaneous bleeding episodes into a single joint within a consecutive 6-month period and were deemed resolved when there had been ≤2 episodes in the joint during the previous 12 months.^10^ An additional exploratory analysis on target joints included investigations into the number of treated spontaneous and traumatic bleeding episodes, assessed by target joint status at baseline (ie, separated into patients with or without target joints at baseline) at the 32-week and 56-week cutoffs.^9^^,^^11^

The number of breakthrough bleeding episodes and hemostatic treatment used to treat bleeds was also investigated as part of exploratory analyses.^12^ The cause, severity, and location of bleeds were reported together with the dose and type of treatment used. Breakthrough bleed severity was classified by the investigator based on the definitions provided in the study protocol (eg, mild/moderate: uncomplicated musculoskeletal bleeds; severe: bleeding episodes that require hospitalization and all life-threatening bleeding episodes).^5^ Mild/moderate breakthrough bleeds were treated as per the bleed management guidance, where the lowest dose of clotting factor concentrate was used in accordance with local labeling while on concizumab and including dosing interval recommendations.^5^

PK/PD assessments consisted of predose concizumab plasma concentration (measured by an enzyme-linked immunosorbent assay), free TFPI concentration (measured by commercially available Asserachom TOTAL TFPI assay, Diagnostica Stago, Asnières-sur-Seine, France), as well as thrombin peak levels (measured by an assay employing low-dose tissue factor in combination with phospholipid as the initiating reagent, with results compared against activity measured in a sample not initiated with tissue factor) at the 56-week cutoff for patients in groups 1 to 4.^5^

Safety assessments reported herein are from the start of the study before the treatment pause until the 56-week cutoff, also including the first 7 weeks during the treatment pause where safety follow-up continued. Overall and specific adverse events (eg, thromboembolic events, hypersensitivity-type reactions, injection site reactions), as well as concizumab anti-drug antibodies were evaluated.^7^ Assessments were performed by specialized laboratories with a clinical trial assay (bridging electrochemiluminescence assay). Anti-drug antibody–positive samples were additionally characterized for in vitro neutralizing activity and titer.

Safety assessments also included exploratory evaluation of minor surgeries that occurred during explorer8.^13^ Minor surgical procedures were permitted during the study and were defined as any invasive operative procedure where only the skin, mucous membranes, or superficial connective tissue was manipulated. The management of these procedures was in accordance with local standards of care and at the discretion of the investigator responsible. Planned/elective major surgery was not permitted during the study. In case of emergent major surgery, temporary concizumab treatment discontinuation was recommended. Local/topical use of antifibrinolytics, as well as single systemic doses, was permitted during surgical procedures after benefit-risk evaluation, and patients undergoing minor surgical procedures continued to receive concizumab prophylaxis during the perioperative period with no change to their concizumab maintenance dose.

### Physical activity and sport activity assessments (32-week cutoff)

Physical and sport activities were assessed at the 32-week cutoff using 2 different methods: the ActiGraph accelerometer (Ametris, LLC, Pensacola, FL) and sport activity ratings; data from neither of these were collected at the 56-week cutoff as they were not within the study parameters at that time point. The ActiGraph accelerometer, worn on the patient’s nondominant wrist, collected data for 2 consecutive weeks before concizumab treatment start (used as baseline values if not previously collected in the noninterventional explorer6 study) and for 8 consecutive weeks at the end of the 32-week cutoff (weeks 24-32 for the concizumab prophylaxis groups or weeks 16-24 for the no prophylaxis group).^14^

Data on sport activity ratings were collected via interviews at baseline and at the end of the main part of the study, where patients were asked about their participation in low- (eg, walking), moderate- (eg, jogging), or high-risk (eg, rugby) sport activities during the previous month.^15^

### Analysis sets

The full analysis set used to investigate efficacy was defined as all patients who had been exposed to concizumab (groups 2-4) on the new dosing regimen or randomized to receive on-demand treatment (group 1) after the treatment restart.

The safety analysis set consisted of all patients who were exposed to concizumab treatment for at least 1 day, either before or after the treatment pause.

The intrapatient analysis set consisted of a subset of patients in the nonrandomized concizumab group 4. Patients included in this analysis set had received their routine, local, standard-of-care prophylaxis (eg, clotting factor concentrate) for at least 24 weeks in the noninterventional explorer6 study and subsequently entered group 4 in the explorer8 study and reached the maintenance dose period on concizumab treatment.

### Statistical analyses

Efficacy, safety, and PK/PD results from the explorer8 56-week cutoff are presented using descriptive statistics; no statistical hypotheses were tested.

Physical activity data collected using the ActiGraph accelerometer (ie, time spent in moderate physical activity and moderate to vigorous physical activity as percentage awake time) were evaluated using the Staudenmayer activity intensity/sedentary classification algorithms (supplemental Methods).^16^ Analysis of covariance with treatment, hemophilia type, and bleeding frequency before screening as fixed effects and the respective baseline value as covariate was used to compare change in physical activity parameters from baseline to the end of the main part of the study between groups 1 (no prophylaxis) and 2 (concizumab prophylaxis).

## Results

### Study population

**Figure 1.**
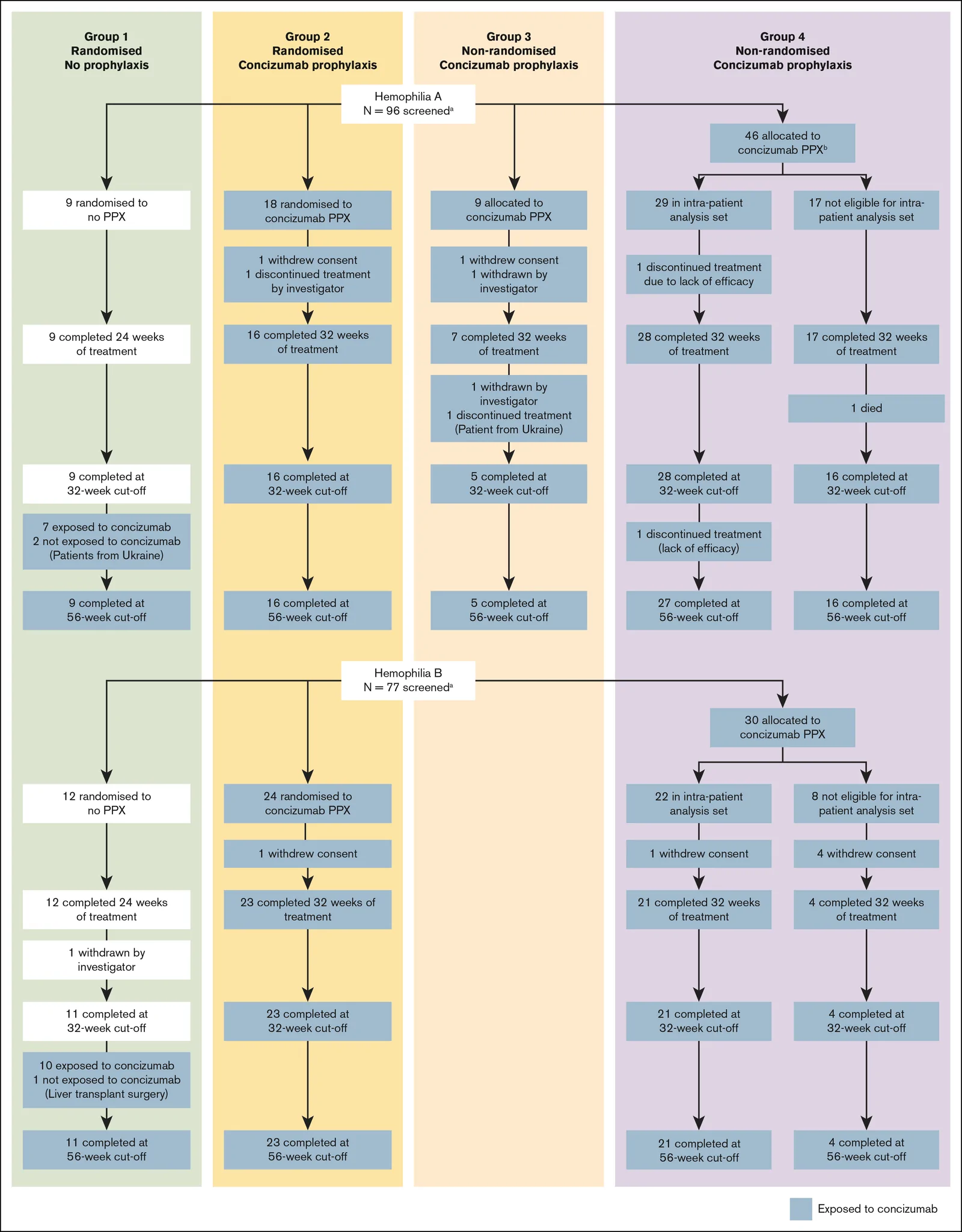
**explorer8 patient disposition.** The study included 4 groups; patients receiving on-demand treatment with no previous exposure to concizumab were randomized 1:2 to group 1 to continue on-demand treatment or to group 2 to receive concizumab prophylaxis. Other patients were allocated to nonrandomized groups 3 and 4 and received concizumab prophylaxis. ^a^Screened before or after the study restart. ^b^Excluding 1 group 1 patient, who withdrew before the study restart. ^c^One patient on concizumab prophylaxis died because of an event of intra-abdominal hemorrhage.^5^ PPX, prophylaxis.

Details on the study population of explorer8 have been previously described.^5^ Overall, the full analysis set comprised 148 patients (n = 82 HA; n = 66 HB; Figure 1). Patients were randomized 1:2 to no prophylaxis in group 1 (n = 9 HA; n = 12 HB) and concizumab prophylaxis in group 2 (n = 18 HA; n = 24 HB), and 85 patients were allocated to concizumab prophylaxis in groups 3 or 4 (n = 55 HA; n = 30 HB). A total of 133 patients (n = 74 HA; n = 59 HB) completed treatment at the 32-week cutoff. After the 32-week cutoff, 17 patients from group 1 switched to concizumab prophylaxis, starting the same dosing regimen as patients in groups 2 to 4. A total of 132 patients (n = 73 HA; n = 59 HB) completed the 56-week cutoff.

### ABRs

The full analysis set used to evaluate efficacy consisted of 148 patients (n = 82 HA; n = 66 HB). Efficacy results excluded 4 patients from group 1 because they were not exposed to concizumab (n = 144 total; n = 80 HA; n = 64 HB). Descriptive results for bleeding episodes in patients with HA/HB from the restart after treatment pause up to the 56-week cutoff were consistent with those previously reported at the 32-week cutoff,^5^^,^^7^ and low bleeding rates were maintained for patients on concizumab prophylaxis (Table 1). Overall, the median annualized bleeding rate (ABR) for treated spontaneous and traumatic bleeding episodes in groups 1 to 4 from the restart after treatment pause up to the 56-week cutoff was 1.7 (interquartile range [IQR], 0.0-4.5) for HA and 2.8 (IQR, 0.0-6.4) for HB (Table 1).

**Table 1.** **Treated spontaneous and traumatic bleeding episodes for patients with HA/HB without inhibitors on concizumab prophylaxis reported at 32- and 56-week cutoffs of the explorer8 study**

	Concizumab prophylaxis (groups 1-4)							
	32-week cutoff				56-week cutoff			
	HA		HB		HA		HB	
Number of patients in full analysis set∗	80		64		80		64	
Patient years of exposure in analysis data set†	79.0		45.2		111.9		71.7	

	**ABR**							
	**Median (IQR)**	**Mean (SD)**	**Median (IQR)**	**Mean (SD)**	**Median (IQR)**	**Mean (SD)**	**Median (IQR)**	**Mean (SD)**
Treated spontaneous and traumatic bleeding episodes	1.6 (0.0-4.9)	4.2 (7.1)	1.7 (0.0-6.4)	6.7 (14.4)	1.7 (0.0-4.5)	3.9 (6.6)	2.8 (0.0-6.4)	6.4 (14.2)
Treated spontaneous bleeding episodes	0.7 (0.0-2.8)	2.7 (5.5)	1.0 (0.0-3.9)	5.2 (13.8)	0.8 (0.0-2.7)	2.4 (5.2)	1.3 (0.0-3.3)	5.0 (13.7)
Treated spontaneous and traumatic joint bleeding episodes	0.7 (0.0-3.5)	3.0 (5.6)	1.3 (0.0-5.3)	4.4 (8.5)	0.9 (0.0-3.6)	2.8 (5.2)	1.6 (0.0-4.6)	4.1 (8.1)
Treated spontaneous and traumatic target joint bleeding episodes	0.0 (0.0-0.0)	0.9 (2.5)	0.0 (0.0-1.0)	2.0 (6.9)	0.0 (0.0-0.0)	0.8 (2.2)	0.0 (0.0-1.5)	2.0 (6.8)
All treated and untreated spontaneous and traumatic bleeding episodes	2.6 (0.6-5.7)	5.4 (8.5)	3.9 (1.3-8.1)	8.1 (14.7)	2.2 (0.5-5.1)	4.9 (7.4)	4.4 (1.1-7.7)	7.6 (14.4)

SD, standard deviation.

∗ The full analysis set was defined as all patients randomized to the new concizumab dosing regimen or on-demand treatment after the treatment pause or allocated to groups 3 or 4 with the new concizumab dosing regimen.

† Analysis data set is defined as patients on treatment without ancillary therapy, excluding data before treatment restart; ancillary therapy was defined as use of factor-containing products not related to treatment of a bleed, except when used for surgery and medical procedures. The 32- and 56-week cutoffs were defined as when all patients in groups 2 to 4 had completed the visit at 32 or 56 weeks or permanently discontinued treatment.^5^^,^^7^

The intrapatient analysis set consisted of a subset of patients from the nonrandomized concizumab group 4 in explorer8 (n = 29 HA; n = 22 HB). Low median ABRs were maintained at the 56-week cutoff in patients with HA/HB who had been on stable prophylaxis previously, consistent with results from the 32-week cutoff.^5^^,^^8^ Median ABR for treated spontaneous and traumatic bleeding episodes in patients on concizumab prophylaxis from the restart after treatment pause up to the 56-week cutoff in the intrapatient analysis set was 1.7 (IQR, 0.5-4.8) for patients with HA and 1.3 (IQR, 0.0-6.4) for patients with HB (Table 2).

**Table 2.** **Treated spontaneous and traumatic bleeding episodes for patients with HA/HB without inhibitors in the intrapatient analysis set reported at 32- and 56-week cutoffs of the explorer8 study**

	HA						HB					
	32-week cutoff				56-week cutoff		32-week cutoff				56-week cutoff	
	Previous PPX		Concizumab PPX		Concizumab PPX		Previous PPX		Concizumab PPX		Concizumab PPX	
Number of patients in intra-patient analysis set∗	29		29		29		22		22		22	
Patient years of exposure	28.9		31.7		44.6		22.1		15.9		25.9	
	**ABR**											
	**Median (IQR)**	**Mean (SD)**	**Median (IQR)**	**Mean (SD)**	**Median (IQR)**	**Mean (SD)**	**Median (IQR)**	**Mean (SD)**	**Median (IQR)**	**Mean (SD)**	**Median (IQR)**	**Mean (SD)**
Treated spontaneous and traumatic bleeding episodes	2.2 (0.8-6.2)	3.8 (4.0)	2.3 (0.0-4.7)	5.7 (10.5)	1.7 (0.5-4.8)	4.8 (9.4)	2.1 (0.9-4.2)	3.1 (3.0)	1.4 (0.0-8.1)	5.2 (10.9)	1.3 (0.0-6.4)	4.7 (10.0)
Treated spontaneous bleeding episodes	1.2 (0.0-2.5)	1.8 (1.8)	0.6 (0.0-2.2)	3.8 (9.7)	0.5 (0.0-2.4)	3.3 (8.9)	0.5 (0.0-2.1)	1.9 (2.9)	0.0 (0.0-3.0)	3.2 (6.1)	0.5 (0.0-3.5)	3.8 (8.9)
Treated spontaneous and traumatic joint bleeding episodes	2.0 (0.0-4.4)	2.6 (2.7)	1.3 (0.0-4.0)	4.5 (9.6)	1.1 (0.0-4.0)	3.8 (8.9)	1.6 (0.0-4.2)	2.7 (3.0)	0.8 (0.0-8.1)	4.0 (7.4)	1.1 (0.0-5.3)	3.5 (6.4)
All treated and untreated spontaneous and traumatic bleeding episodes	3.0 (1.0-6.2)	4.0 (4.0)	2.6 (0.7-6.0)	6.1 (10.4)	2.4 (0.5-5.3)	5.1 (9.3)	2.1 (1.4-4.2)	3.4 (3.5)	2.1 (0.0-8.7)	6.1 (11.3)	2.5 (0.7-7.6)	5.9 (10.4)

PPX, prophylaxis; SD, standard deviation.

∗ The intrapatient analysis set consisted of a subset of patients from the nonrandomized concizumab group 4 in explorer8 who were on previous stable factor prophylaxis of ≥24 weeks in the noninterventional explorer6 (observational study [ClinicalTrials.gov identifier: NCT03741881]). Time frame for previous prophylaxis in explorer6: from the point in time where prophylaxis was stable (after an initial period on prophylaxis treatment of ≥24 weeks) until the end of study. Time frame for concizumab prophylaxis in explorer8: from the point in time when concizumab maintenance dose was confirmed until the 32- or 56-week cutoffs. The 32- and 56-week cutoffs were defined as when all patients in groups 2 to 4 had completed the visit at 32 or 56 weeks or permanently discontinued treatment.^8^

### Target joints

A total of 63 patients with HA/HB (45/108 adults [41.7%]; 18/36 adolescents [50.0%]) had at least 1 target joint at baseline. Most target joints were reported in the knee, elbow, or ankle, with a few also reported in the hip or shoulder (supplemental Table 1). At the 56-week cutoff, 69 out of 80 target joints (86.3%) were resolved, with most target joints resolving within 12 months after the start of concizumab treatment (median, 12.0 months [IQR, 12.0-12.0]).

Low median ABRs for both treated spontaneous and traumatic joint and target joint bleeding episodes were maintained at the 56-week cutoff in participants in groups 1 to 4 receiving concizumab prophylaxis. The median ABRs for treated spontaneous and traumatic bleeding episodes from the restart after treatment pause until the 56-week cutoff for target joints were 0.0 (IQR, 0.0-0.5) for adults and 0.0 (IQR, 0.0-1.3) for adolescents and 0.9 (IQR, 0.0-3.6) for adults and 1.6 (IQR, 0.6-6.0) for adolescents for joint bleeding episodes (supplemental Table 1).

Further exploratory analysis investigated the number of treated overall spontaneous and traumatic bleeding episodes assessed by the presence or absence of target joints at baseline. Descriptive statistics on median ABRs for treated spontaneous and traumatic bleeding episodes during concizumab prophylaxis (groups 1-4) showed that ABRs were low at 32 weeks for both patients with (3.2 [IQR, 0.0-6.4]) and without (1.6 [IQR, 0.0-4.5]) target joints at baseline. Median ABRs remained low at 56 weeks regardless of target joint status at baseline, where patients with target joints at baseline had a median ABR of 2.9 (IQR, 0.6-6.4) and 1.0 (IQR, 0.0-4.4) for patients without target joints at baseline (supplemental Table 2).

### Breakthrough bleeding episodes

Most breakthrough bleeds in patients on concizumab prophylaxis at the 56-week cutoff were spontaneous (53.5% in HA; 63.8% in HB), were mild/moderate in severity (96.9% HA; 95.8% HB), and occurred mostly in the joints (68.5% in HA; 74.6% in HB) (Table 3). For the 80 patients with HA on concizumab, 23 patients (28.8%) had zero treated bleeding episodes up until the 56-week cutoff. For the 64 patients with HB on concizumab, 14 patients (21.9%) had zero treated bleeding episodes up until the 56-week cutoff.

**Table 3.** **Summary of breakthrough bleeds for patients with HA/HB without inhibitors on concizumab prophylaxis reported at 56-week cutoff of the explorer8 study**

	HA		HB	
No. of patients in analysis set	80		64	
No. of patients with treated bleeding episodes, n (%)	56 (70.0)		48 (75.0)	
No. of patients with zero treated bleeds, n (%)∗	23 (28.8)		14 (21.9)	
Total no. of treated bleeding episodes, n	355		307	

	**No. of patients, n (%)**	**No. of bleeding episodes, n (%)**	**No. of patients, n (%)**	**No. of bleeding episodes, n (%)**

**Cause of bleed**				
Spontaneous	47 (58.8)	190 (53.5)	39 (60.9)	196 (63.8)
Traumatic	41 (51.3)	159 (44.8)	29 (45.3)	106 (34.5)
Surgical	5 (6.3)	6 (1.7)	5 (7.8)	5 (1.6)
**Severity of bleed**†				
Mild/moderate	56 (70.0)	344 (96.9)	46 (71.9)	294 (95.8)
Severe	3 (3.8)	11 (3.1)	8 (12.5)	13 (4.2)
**Location of bleed**‡				
Joint	53 (66.3)	259 (68.5)	43 (67.2)	247 (74.6)
Target joint	18 (22.5)	73 (19.3)	23 (35.9)	89 (26.9)
Muscular	21 (26.3)	47 (12.4)	12 (18.8)	44 (13.3)

n, number of patients.

∗ Patients who did not complete 24 weeks without permanent treatment discontinuation were counted as not fulfilling this, and all treated bleeds with either of the 4 causes (spontaneous, traumatic, surgical, or missing) were taken into account.

† The investigator determined the classification of bleed severity as follows: mild/moderate, uncomplicated musculoskeletal, mucosal, or subcutaneous bleeds; severe, bleeds that required hospitalization or were life-threatening.

‡ A bleeding episode that occurred in multiple locations was counted in all these locations; only the 3 most frequent sites of bleeds are included.^12^

Breakthrough bleeds of mild/moderate severity were mostly treated with 1 injection of FVIII (HA) or FIX (HB) (supplemental Table 3). Bleed treatment dose was given according to label and as per the bleed management guidance (for first dose: 20 IU/kg for FVIII standard/extended half-life; 30 IU/kg for FIX standard/extended half-life),^5^ where the median consumption per injection for mild/moderate bleeds was 20 IU/kg for HA (number of bleeds treated with FVIII = 335) and 30 IU/kg for HB (number of bleeds treated with FIX = 289). The median consumption per injection for severe bleeds was 21 IU/kg for HA (number of bleeds treated with FVIII = 11) and 29 IU/kg for HB (number of bleeds treated with FIX = 13).

### PK and PD

**Figure 2.**
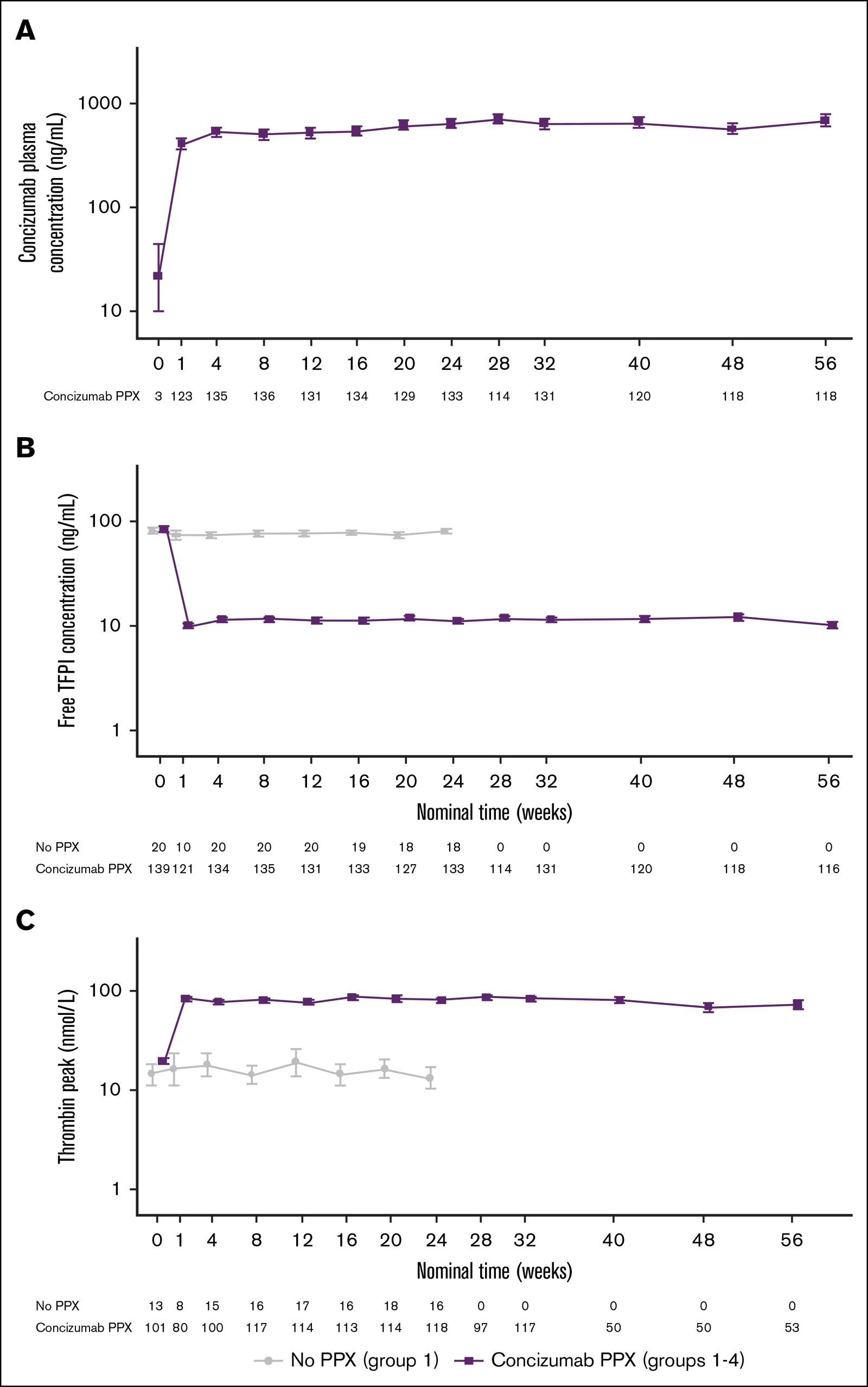
**PK and PD measures for patients with HA/HB without inhibitors reported at the 56-week cutoff of the explorer8 study.** Geometric mean (A) predose trough concizumab plasma concentrations, (B) predose free TFPI in plasma, and (C) thrombin peak. Error bars represent ± standard error of the geometric mean.^7^ PPX, prophylaxis.

At the 56-week cutoff, concizumab administration continued to correspond with free TFPI suppression and normalization of thrombin generation, consistent with the 32-week cutoff results. The geometric mean predose trough concizumab plasma concentration for patients on concizumab prophylaxis (groups 1-4) remained similar to the measurement after 24 weeks of treatment (642.1 ng/mL) at the 56-week cutoff (682.7 ng/mL; geometric coefficient of variation, 239.1%) (Figure 2A). Concizumab exposure range was stable over time until the 56-week cutoff. Including patients in group 1 who switched to concizumab, the maintenance dose was increased to 0.25 mg/kg in 35 (25.4%) patients, decreased to 0.15 mg/kg in 10 (7.2%) patients, and maintained at 0.20 mg/kg in 93 (67.4%) patients based on concizumab plasma concentration measured after week 4 of concizumab treatment. Six patients (n = 2 HA; n = 4 HB) withdrew from the study and did not have a maintenance dose reported.

The geometric mean predose free TFPI concentration decreased from 84.2 ng/mL at baseline to 11.1 ng/mL at week 24 for patients on concizumab prophylaxis (groups 1-4) (Figure 2B). After the initial decrease, levels remained stable until the 56-week cutoff (geometric mean, 10.2 ng/mL). For patients on no prophylaxis (group 1) during the main part of the study, free TFPI remained at levels similar to baseline.

At week 24, the geometric mean predose thrombin peak levels increased from 21.1 nmol/L at baseline to 81.3 nmol/L (within normal range) for patients on concizumab prophylaxis (groups 1-4) and remained stable over time at levels similar to baseline for patients on no prophylaxis (group 1) (Figure 2C). After the initial increase, the levels remained stable over time until week 56, where the geometric mean level was 72.8 nmol/L for patients on concizumab prophylaxis.

### Safety

The safety analysis set comprised 151 patients exposed to at least 1 dose of concizumab (n = 87 HA; n = 64 HB).

Descriptive safety results at the 56-week cutoff (Table 4) were consistent with those at the 32-week cutoff.^5^^,^^7^ No thromboembolic events were reported after treatment restart until the 56-week cutoff, and no events of disseminated intravascular coagulation, thrombotic microangiopathy, or hypersensitivity-type reactions were reported during the entire study. Safety data until the 32-week cutoff have been reported previously.^5^ No additional patients reported adverse events with fatal outcomes, adverse events that led to treatment withdrawal, or thromboembolic events between the 32- and 56-week cutoffs; 8 additional serious adverse events occurred in 8 patients (all reported as unlikely related to concizumab and recovered). Most events (7 events; 87.5%) did not lead to a change in concizumab dose. One event (cartilage injury; recovered) led to concizumab treatment interruption because of the patient undergoing knee replacement surgery. For this patient, concizumab treatment was paused 72 hours before surgery and restarted ∼1.5 months after surgery. From the 32- to the 56-week cutoff, 3 patients reported 6 injection site reactions, which were mild and did not lead to drug withdrawal. The rate and type of injection site reactions were unchanged.

**Table 4.** **Adverse events reported for patients with HA/HB without inhibitors on concizumab prophylaxis at the 56-week cutoff of the explorer8 study**

	Concizumab prophylaxis (groups 1-4)∗			
	HA/HB			
	Patients		Events	Rate
	n	%	n	Events/PYE
No. of patients in safety analysis set†	151	100		
Patient years of exposure	193.6			
Total adverse events	118	78.1	528	2.7
Serious adverse events‡	20	13.2	30	0.2
Fatal adverse events§	1	0.7	1	0.0
Adverse events leading to drug withdrawal||	6	4.0	8	0.0
**Adverse events of special interest**				
Thromboembolic events¶	2	1.3	4	0.0
Thromboembolic events after treatment restart up to 56-week cutoff#	0			
**Adverse events with additional data collection**				
Hypersensitivity-type reactions	0			
Injection site reactions∗∗	26	17.2	46	0.2
Medication errors	4	2.6	5	0.0

n, number of patients or events; PYE, patient years of exposure.

∗ Although patients were on concizumab prophylaxis, including initial dosing regimen before the treatment pause (134 patients in groups 2-4 were exposed to concizumab before and/or after treatment pause; 17 patients in group 1 switched to concizumab prophylaxis after ≥24 weeks of treatment in the study).

† The safety analysis set was defined as all patients exposed to concizumab prophylaxis or randomized to on-demand treatment before and after the treatment pause.

‡ Eight serious adverse events were reported in 8 patients between the 32- and 56-week cutoffs, all unlikely related to concizumab and assessed as “recovered,” comprising 1 event each of ureterolithiasis, ligament sprain, abdominal pain, traumatic hematoma, cartilage injury, gastroenteritis rotavirus, renal hematoma, and pneumonia.

§ Before the 32-week cutoff, 1 patient with HA treated with concizumab with a long-standing history of hypertension experienced a severe adverse event with fatal outcome (intra-abdominal hemorrhage), possibly related to concizumab, as judged by the investigator. Event onset was unknown because the patient was found deceased. Autopsy showed no evidence of thromboembolism, vascular injury, or trauma; liver and spleen were intact. Concomitant medications comprised a β1-adrenergic receptor blocker. Of note, the patient was exposed to concizumab in phase 2 of the explorer5 study (ClinicalTrials.gov identifier: NCT03196297) before continuing in explorer8, with no reported bleeds in either study up to this point.

‖ Drug withdrawal was defined as when treatment was permanently discontinued but does not necessarily indicate that the patient withdrew from the study. Adverse events leading to drug withdrawal included deep venous thrombosis, pulmonary embolism, and superficial vein thrombosis in 1 patient; acute myocardial infarction in 1 patient; craniocerebral injury in 1 patient; cardiac failure in 1 patient; intra-abdominal hemorrhage in 1 patient; and injection site pain in 1 patient.

¶ Nonfatal thromboembolic events in 2 patients were reported (deep venous thrombosis, pulmonary embolism, and superficial vein thrombosis in 1 patient, and acute myocardial infarction in 1 patient).

# Treatment was paused from March 2020 to August 2020 because of nonfatal thromboembolic events and restarted after implementation of risk mitigations, including a new concizumab dosing regimen and guidance on mild and moderate breakthrough bleed management with lowest dose of factor product while on concizumab.

∗∗ Six injection site reactions were reported in 3 patients between the 32- and 56-week cutoff and comprised injection site reaction (2), injection site pain (1), application site pain (1), injection site mass (1), and injection site rash (1).^7^

### Anti-concizumab antibodies

At the 56-week cutoff, low-titer anti-concizumab antibodies were detected in 13 patients with HA (14.9%; 1 additional patient since the 32-week cutoff) and 8 patients with HB (12.5%; 2 additional patients since the 32-week cutoff). One patient who tested positive for low-titer anti-concizumab antibody with in vitro neutralizing effect reported temporarily reduced concizumab concentration and restored free TFPI. The observed changes in PK/PD were unlikely associated with anti-concizumab antibody presence because the decrease in concizumab concentration and the restoration of free TFPI continued after titer values decreased and the patient tested negative for in vitro neutralizing anti-concizumab antibodies. Moreover, the patient missed 5 consecutive concizumab doses before sample collection, and the decrease in concizumab concentration or the increase in free TFPI was not accompanied by any significant worsening in bleeding patterns or occurrence of immunogenicity-related adverse events in this patient. Overall, there was no impact of anti-concizumab antibodies on bleeding episodes, adverse events (injection site and hypersensitivity reactions), or PK/PD.

### Surgery

Of the 151 patients exposed to concizumab, 19 patients (n = 9 HA; n = 10 HB) underwent 24 minor surgical procedures, of which 15 were dental procedures, including dental extractions, removal of dental deposits by ultrasonic scaler, tooth implants, braces setting, and procedures related to dental cavities. Other minor surgical procedures included colonoscopy, arthrodesis, procedure related to pyoderma of the buttock, urethral augmentation after acute urinary retention, and hyaluronic acid infiltration into ankle related to arthropathy. No drug discontinuation was required for minor surgeries, and factor products were used to manage patients according to local standards of care. Further information on hemostatic agents used during minor surgeries are detailed elsewhere.^13^^,^^17^

### Physical and sport activity (32-week cutoff)

Overall, 41 of 63 patients randomized to groups 1 and 2 contributed to the exploratory physical activity analyses using data from the ActiGraph accelerometer at the 32-week cutoff. No differences in physical activity (expressed as % of awake time) measured by the ActiGraph accelerometer were observed in patients with HA/HB on concizumab prophylaxis (group 2) vs no prophylaxis (group 1) for any level of activity (supplemental Table 4).

Sport activity ratings collected through interviews showed that the proportion of patients participating in sport activities at various risk levels at the end of the 32-week cutoff was comparable to the baseline values for the corresponding patients (supplemental Figure 2).

## Discussion

Descriptive efficacy results at the phase 3 explorer8 56-week cutoff confirmed the effectiveness of concizumab as a once-daily subcutaneous prophylactic agent for patients with HA/HB without inhibitors, as low ABRs were maintained with longer-term use. The study also included a nonrandomized, intrapatient comparison of concizumab to previous stable factor prophylaxis. Descriptive efficacy results for longer-term concizumab use in the intrapatient analysis set also showed that low ABRs were maintained at the 56-week cutoff.

Results obtained at the explorer8 56-week cutoff reflected those seen at the 32-week cutoff, where daily subcutaneous concizumab prophylaxis compared with no prophylaxis (on-demand treatment) effectively reduced the number of treated spontaneous and traumatic bleeding episodes in patients with HA/HB without inhibitors by 86% and 79%, respectively.^5^ Furthermore, the reduction in bleeding episodes during concizumab prophylaxis observed in explorer8 was consistent with that observed in the phase 3 explorer7 study in patients with HA/HB with inhibitors.^6^

Results from the exploratory analyses on target joint and joint bleeding episodes, as well as breakthrough bleeds, also showed that a low bleeding rate was maintained with longer-term concizumab use, regardless of the presence or absence of target joints at baseline, with breakthrough bleeds managed according to label and protocol recommendations. Most breakthrough bleeds in patients receiving concizumab prophylaxis occurred in joints and were of mild/moderate severity. Importantly, most mild/moderate breakthrough bleeds were safely treated and resolved with a single injection of factor products according to label, indicating no new safety signals. However, the cause of most breakthrough bleeds was recorded as “spontaneous,” which was unexpected; a small number of patients with extreme ABRs may have contributed to this result. Recurring joint and muscle bleeds are debilitating to patients because they lead to long-term damage such as hemophilic arthropathy, causing pain and a decrease in quality of life.^18^ Although joint bleeding episodes were still reported, their reduced frequency with concizumab prophylaxis compared with no prophylaxis, as well as treatment with a single dose of factor, reflects successful hemostatic protection achieved with longer-term concizumab use. Concizumab is therefore a beneficial addition to the current hemophilia treatment landscape, especially for patients with HB who have limited approved subcutaneous treatment options.^4^

Consistent with results at the 32-week cutoff for patients with and without inhibitors,^5^^,^^6^ concizumab PK/PD, as assessed by measurements of concizumab plasma concentration, free TFPI concentration, and thrombin peak levels, remained stable over time until the 56-week cutoff.

Past studies of emicizumab have shown that nonfactor therapies may have a higher risk of thromboembolic events compared with replacement therapies because of drug-drug interaction.^19^ In addition, long-term safety data are limited for nonfactor therapies, in contrast to factor replacement. Although thromboembolic events were seen earlier in the concizumab explorer studies, no further events were reported after the treatment pause until the 56-week cutoff. Furthermore, no thrombotic microangiopathy or disseminated intravascular coagulation was reported during any concizumab clinical studies. Based on the current data, there are no new safety findings to note, demonstrating overall safety of concizumab with longer-term use if the concizumab dosing regimen and bleed treatment guidance are followed.

Other adverse events to note were injection site reactions and anti-concizumab antibodies reported between the 32- and 56-week cutoffs. Reported injection site reactions were mild in severity and did not change the overall rate vs the 32-week cutoff, whereas there was also no change in the type of reactions reported. It is important to note that anti-drug antibody formation is common with monoclonal antibody treatment.^20^ Regarding the patients who tested positive for anti-concizumab antibodies since the 32-week cutoff (1 patient with HA and 2 patients with HB), all antibodies were low-titer, with no impact on bleeding patterns or adverse events, and only 1 patient experienced temporarily reduced concizumab levels and restored free TFPI, similar to what had been reported at the 32-week cutoff.^5^

Physical activity improvements were seen in explorer7 in patients with HA/HB with inhibitors on concizumab vs no prophylaxis.^14^ However, no differences in physical activity were observed in the current study in patients without inhibitors. Of note, explorer8 coincided with the COVID-19 pandemic, and the resulting lockdown periods may have influenced patients’ physical activity. Results for other patient-reported outcomes in this cohort were collected for the 32-week cutoff analysis and have, nevertheless, demonstrated improvements in health-related quality of life, a reduction in treatment burden with concizumab, and a preference for concizumab over previous treatment among most patients.^21^^,^^22^ More specific results on physical functioning using the Patient Global Impression of Severity on physical functioning also illustrated that, among patients on concizumab who responded to the questionnaire, 27.5% reported their level of physical functioning as “very good,” 46.1% as “good,” 20.6% as “fair,” 3.9% as “poor,” and 2.0% as “very poor.” These results provide support for concizumab prophylaxis compared with no prophylaxis as a treatment option for patients with HA/HB with and without inhibitors.^21^^,^^22^

As previously noted,^5^^,^^6^ limitations in this study include the open-label design and the nonrandomized nature of the intrapatient analysis set. Limitations more specific to the 56-week cutoff analyses were the exploratory nature of the surgical data assessments, target joint analysis, ABR in patients based on target joints at baseline, breakthrough bleeds, and their management, which means results should be interpreted with caution.

Overall, the explorer8 56-week cutoff results showed that longer-term daily concizumab prophylaxis was effective in maintaining low bleeding rates and was considered safe and well tolerated in patients with HA/HB without inhibitors. Results were consistent with the 32-week cutoff and support concizumab as a promising subcutaneous prophylaxis treatment, especially for patients with HB. To date, longer-term data for nonfactor therapies are limited. Hemophilia is a lifelong journey, with long-term treatment required by the affected population; reports on longer-term concizumab use are timely and of clinical importance as a crucial part of evaluating the treatment, especially when it represents a novel mode of action.

Conflict-of-interest disclosure: G.Y. reports consulting fees from Alnylam, ASC Biotherapeutics, Bayer, BioMarin, CSL Behring, Genentech/Roche, Hema Biologics, Hemab, Novo Nordisk, Octapharma, Pfizer, Sanofi Genzyme, Spark, and Takeda; and funds for research support from Sanofi. P.A.’s institution has received project-based funding through research agreements from Novo Nordisk and Sanofi. R.B.F. and M.F.M.G. report employment at Novo Nordisk A/S. A.K.C.C. reports clinical trial support from Bayer, Novo Nordisk, Roche, Sanofi, and Takeda; and honoraria from Bayer, Roche, and Sanofi. P.C. reports grants from BioMarin, Freeline, Novo Nordisk, Pfizer, and Sobi; research support from Roche, Freeline, Novo Nordisk, Apcintex, and Sobi; and advisory and/or speaker support from Bayer, Apcintex, CSL Behring, Chugai, Metagenomics, Novo Nordisk, Pfizer, Sanofi, Sobi, Takeda, and Roche. H.E. reports institutional grants for research and studies from Bayer Vital and CSL Behring; and honoraria for lectures or consultancy from Bayer Vital, BioMarin, CSL Behring, Novo Nordisk, Pfizer, Roche, and Sobi. T.M. is a member of advisory boards for Takeda, Bayer, Novo Nordisk, Chugai, and Pfizer; reports educational and investigational support from Chugai and Novo Nordisk; and honoraria from Takeda, Bayer, Sanofi, Chugai, CSL Behring, JB Pharma, KMB Pharma, Novo Nordisk, and Pfizer. S.Š.T. reports speaker fees from or participation in advisory boards for Novo Nordisk, Roche, Bayer AG, Takeda, Octapharma, Sobi, and Amgen. A.T. reports consulting/advisory roles with Novo Nordisk, Pfizer, Roche, Sobi, and Takeda; research funding from Octapharma and Novo Nordisk; and travel, accommodation, and expenses support from Novo Nordisk and Sobi. J.W. reports research funding or speaker fees from or participation in advisory boards for Alnylam, Amgen, AstraZeneca, Bayer AG, CSL Behring, LFB, Novartis, Novo Nordisk, Octapharma, Pfizer, Roche, Sanofi, Siemens, Sobi, Takeda, and Werfen. J.A. reports research funding from Sobi/Sanofi and CSL Behring; and speaker fees from or participation in advisory boards for Novo Nordisk, Sobi, CSL Behring, Bayer, Octapharma, Pfizer, BioMarin, Roche, and Takeda. The remaining authors declare no competing financial interests.

## Supplementary Material

Supplemental Methods, Tables, Figures, and References
