# Supplementary material for: Concizumab in patients with hemophilia A or B without inhibitors: 56-week cutoff results of the phase 3 explorer8 study
Source: Blood Adv. 2026 Jun 26;10(17):6032–42. doi: 10.1182/bloodadvances.2026019931 (PMC13558241; doi:10.1182/bloodadvances.2026019931)
Supplement: Supplementary file 1 [file BLOODA_ADV-2026-019931-mmc1.pdf]

SUPPLEMENTARY MATERIALS

Concizumab in patients with hemophilia A/B without inhibitors: 56-week cut-off results of the phase 3 explorer8 study

Longer-term (≥1 year) concizumab in hemophilia

Authors:

Guy Young,[1*] Pantep Angchaisuksiri,[2] Shashikant Apte,[3] Renée Brown Frandsen,[4] Anthony KC Chan,[5] Pratima Chowdary,[6] Hermann Eichler,[7] Chuhl Joo Lyu,[8] Maria Fernanda Martinez Garcia,[4] Tadashi Matsushita,[9] Sonata Šaulytė Trakymienė,[10] Hyuen Tran,[11,12] Alice Trinchero,[13] Jerzy Windyga,[14] and Jan Astermark[15]

Affiliations:

[1]Hemostasis and Thrombosis Center, Cancer and Blood Disorders Institute, Children's Hospital of Los Angeles, Keck School of Medicine, University of Southern California, Los Angeles, CA, USA; [2]Division of Hematology, Department of Medicine, Ramathibodi Hospital, Mahidol University, Bangkok, Thailand; [3]Department of Hematology, Sahyadri Specialty Hospitals, Pune, India; [4]Novo Nordisk A/S, Søborg, Denmark; [5]Department of Pediatrics, McMaster University, Hamilton, Ontario, Canada; [6]Katharine Dormandy Haemophilia and Thrombosis Centre, Royal Free London NHS Foundation Trust, London, UK; [7]Institute of Clinical Hemostaseology and Transfusion Medicine, Saarland University and University Hospital, Homburg, Germany; [8]Department of Paediatric Haematology-Oncology, Yonsei Cancer Center, Severance Hospital, Yonsei University College of Medicine, Seoul, South Korea; [9]Department of Transfusion Medicine, Nagoya University Hospital, Nagoya, Japan; [10]Clinic of Children's Diseases, Faculty of Medicine, Vilnius University Hospital Santaros Klinikos, Vilnius University, Vilnius, Lithuania; [11]Ronald Sawyers Haemophilia Centre, The Alfred Hospital, Melbourne, Victoria, Australia; [12]Australian Centre for Blood Diseases, Monash University, Melbourne, Victoria, Australia; [13]Clinic for Medical Oncology and Haematology, University Hospital Zurich, Zurich, Switzerland; [14]Department of Hemostasis Disorders and Internal Medicine, Laboratory of Hemostasis and Metabolic Diseases, Institute of Hematology and Transfusion Medicine, Warsaw, Poland; [15]Institution of Translational Medicine, Lund University, and Department of Hematology Oncology and Radiation Physics, Skåne University Hospital, Malmö, Sweden.

***Corresponding author**

Name: Prof. Guy Young

Address: Children's Hospital Los Angeles, 4650 Sunset Blvd, Mail Stop 54, Los Angeles, CA 90027, United States

Email address: gyoung@chla.usc.edu

Phone number: +1 323-361-5507

Table of contents

Supplementary methods 3
Physical activity analyses 3

Supplementary tables 4
Table S1. Location of target joints and median ABR at baseline reported for adolescent and adult patients with hemophilia A or B without inhibitors on concizumab prophylaxis at the 56-week cut-off of the explorer8 study 4
Table S2. Median ABR for spontaneous and traumatic bleeding episodes, joint bleeding episodes and target joint bleeding episodes in patients with hemophilia A or B without inhibitors on concizumab prophylaxis with and without target joints at baseline reported at 32- and 56-week cut-offs of the explorer8 study 5
Table S3. Summary of breakthrough bleed management for patients with hemophilia A or B without inhibitors on concizumab prophylaxis reported at 56-week cut-off of the explorer8 study 6
Table S4. Change in physical activity parameters measured using the ActiGraph™ accelerometer and difference estimates between patients on concizumab prophylaxis (group 2) compared with no prophylaxis (group 1) from baseline to the 32-week cut-off in the explorer8 study 7
Figure S1. explorer8 study design 8
Figure S2: Summary of sport activity ratings at (A) baseline for all patients in the full analysis set, (B) baseline for patients with physical activity ratings data at the 32-week cut-off, and (C) corresponding data at the 32-week cut-off in the explorer8 study...... 9
References 10

Supplementary methods

Physical activity analyses

Physical activity assessed with the ActiGraph™ accelerometer was analyzed at the 32-week cut-off. The Staudenmayer activity intensity/sedentary classification algorithms were used to evaluate the endpoints for classification of levels of physical activity for the ActiGraph™ data (i.e., time spent in moderate physical activity and moderate-to-vigorous physical activity as percentage awake time).[1] Wear time and sleep time were derived from the physical activity data using the wear and sleep algorithms (developed on adults), respectively. All derivations concerning physical activity levels were based on detected wear time not classified as sleep (awake time: time with wear but not sleep; non-wear time: records where the wear status was 'no'). The hierarchy for the algorithms prioritized sleep algorithm first, and physical activity endpoint algorithm second, meaning that one minute cannot be categorized according to an endpoint algorithm if it had already been classified as sleep.

Supplementary tables

Table S1. Location of target joints and median ABR at baseline reported for adolescent and adult patients with hemophilia A or B without inhibitors on concizumab prophylaxis at the 56-week cut-off of the explorer8 study

<table>
<tr><th colspan="2" rowspan="2"></th><th colspan="4">Hemophilia A or B without inhibitors on concizumab prophylaxis (groups 1–4)</th></tr>
<tr><th colspan="2">Adolescents</th><th colspan="2">Adults</th></tr>
<tr><td colspan="2">Number of patients in the analysis set</td><td colspan="2">36</td><td colspan="2">108</td></tr>
<tr><td colspan="2">Number of patients having at least one target joint at baseline</td><td colspan="2">18</td><td colspan="2">45</td></tr>
<tr><th colspan="6">ABR</th></tr>
<tr><td rowspan="2">Treated spontaneous and traumatic joint bleeding episodes</td><td>Median (IQR)</td><td colspan="2">1.6 (0.6; 6.0)</td><td colspan="2">0.9 (0.0; 3.6)</td></tr>
<tr><td>Mean (SD)</td><td colspan="2">5.3 (8.6)</td><td colspan="2">2.8 (5.8)</td></tr>
<tr><td rowspan="2">Treated spontaneous and traumatic target joint bleeding episodes</td><td>Median (IQR)</td><td colspan="2">0.0 (0.0; 1.3)</td><td colspan="2">0.0 (0.0; 0.5)</td></tr>
<tr><td>Mean (SD)</td><td colspan="2">1.4 (3.0)</td><td colspan="2">1.3 (5.3)</td></tr>
<tr><th colspan="2">Target joint location</th><th>Number of patients</th><th>Number of target joints</th><th>Number of patients</th><th>Number of target joints</th></tr>
<tr><td colspan="2">Ankle</td><td>7</td><td>9</td><td>20</td><td>22</td></tr>
<tr><td colspan="2">Elbow</td><td>8</td><td>9</td><td>19</td><td>23</td></tr>
<tr><td colspan="2">Knee</td><td>7</td><td>10</td><td>21</td><td>29</td></tr>
<tr><td colspan="2">Hip</td><td>1</td><td>1</td><td>2</td><td>2</td></tr>
<tr><td colspan="2">Shoulder</td><td>0</td><td>0</td><td>3</td><td>3</td></tr>
</table>

Target joints at baseline were evaluated in all patients and were reported per age group for patients having at least one target joint (ankle, elbow, hip, knee, and shoulder). ABR, annualized bleeding rate; IQR, interquartile range; SD, standard deviation.[2]

Table S2. Median ABR for spontaneous and traumatic bleeding episodes, joint bleeding episodes and target joint bleeding episodes in patients with hemophilia A or B without inhibitors on concizumab prophylaxis with and without target joints at baseline reported at 32- and 56-week cut-offs of the explorer8 study

		Hemophilia A or B without inhibitors on concizumab prophylaxis (groups 1–4)			
		With target joints at baseline		Without target joints at baseline	
		32-week cut-off	56-week cut-off	32-week cut-off	56-week cut-off
**Number of patients in the analysis set**		67		77	
**Median ABR (interquartile range)**					
All treated and untreated spontaneous and traumatic bleeding episodes	Median (IQR)	3.3 (0.7; 7.8)	3.6 (1.1; 7.3)	3.0 (0.6; 5.9)	2.4 (0.9; 6.5)
	Mean (SD)	6.9 (10.6)	6.4 (10.2)	6.3 (12.6)	5.8 (12.0)
Treated spontaneous and traumatic bleeding episodes	Median (IQR)	3.2 (0.0; 6.4)	2.9 (0.6; 6.4)	1.6 (0.0; 4.5)	1.0 (0.0; 4.4)
	Mean (SD)	6.0 (10.0)	5.7 (9.7)	4.7 (11.9)	4.4 (11.5)
Treated spontaneous and traumatic joint bleeding episodes	Median (IQR)	1.9 (0.0; 6.4)	2.7 (0.0; 4.9)	0.0 (0.0; 1.9)	0.5 (0.0; 1.9)
	Mean (SD)	4.9 (8.3)	4.7 (8.0)	2.4 (5.6)	2.3 (5.1)
Treated spontaneous and traumatic target joint bleeding episodes	Median (IQR)	0.0 (0.0; 3.3)	0.9 (0.0; 3.4)	N/A	N/A
	Mean (SD)	3.0 (7.0)	2.9 (6.8)	N/A	N/A

Target joint is defined as having three or more spontaneous bleeds into a single joint within a consecutive 6-month period. The 32- and 56-week cut-offs were defined as when all patients had completed the visit after 32 or 56 weeks respectively, or permanently discontinued treatment. ABR, annualized bleeding rate; IQR, interquartile range; N/A, not applicable; SD, standard deviation.[3]

Table S3. Summary of breakthrough bleed management for patients with hemophilia A or B without inhibitors on concizumab prophylaxis reported at 56-week cut-off of the explorer8 study

	Hemophilia A		Hemophilia B	
	**Mild/moderate bleed**	**Severe bleed**	**Mild/moderate bleed**	**Severe bleed**
**Factor VIII (FVIII)**				
Number of bleeds treated with FVIII, n (%)	335 (100.0)	11 (100.0)	-	
1 injection	242 (72.2)	0	-	
2 injections	54 (16.1)	0	-	
≥3 injections	39 (11.6)	11 (100.0)	-	
**Factor IX (FIX)**				
Number of bleeds treated with FIX, n (%)	-		289 (100.0)	13 (100.0)
1 injection	-		213 (73.7)	9 (69.2)
2 injections	-		43 (14.9)	0
≥3 injections	-		33 (11.4)	4 (30.8)

For some bleeding episodes, the dosing information for the associated treatment was missing; and in some cases, a bleeding episode was treated with more than one product.[4] FIX, factor IX; FVIII, factor VIII.

Table S4. Change in physical activity parameters measured using the ActiGraph™ accelerometer and difference estimates between patients on concizumab prophylaxis (group 2) compared with no prophylaxis (group 1) from baseline to the 32-week cut-off in the explorer8 study

	**No prophylaxis n=12 (group 1)**	**Concizumab prophylaxis n=29 (group 2)**	**Difference estimate**	**P-value**
**Moderate activity (% awake time) [95% CI]**	-0.3 [-2.7; 2.1]	0.4 [-1.1; 2.0]	0.8 [-2.1; 3.7]	0.6
**Moderate-to-vigorous activity (% awake time) [95% CI]**	-0.6 [-3.3; 2.2]	0.7 [-1.0; 2.5]	1.3 [-2.0; 4.5]	0.4
**Vigorous activity (% awake time) [95% CI]**	-0.2 [-0.8; 0.4]	0.3 [-0.1; 0.6]	0.5 [-0.2; 1.2]	0.2

The data presented here reflect the observation period in which patients were exposed to either on-demand treatment (no prophylaxis) or the new concizumab dosing regimen following the treatment pause due to thromboembolic events in patients in the two phase 3 studies (explorer7/8). Observation time during the initial concizumab dosing regimen and data collected after permanent treatment discontinuation are not included. Although 63 patients with hemophilia A/B without inhibitors were randomized into groups 1 (n=21) and 2 (n=42), a total of 22 patients did not contribute to the physical activity exploratory analysis in explorer8 (n=9 from group 1 and n=13 from group 2); three patients were withdrawn, one patient discontinued treatment before 24 weeks, and 18 patients did not have data from baseline and/or the end of the main part of the study. The reasons included broken device, device not being able to transfer data, patients forgetting to wear the device. CI, confidence interval.[5]

Figure S1. explorer8 study design

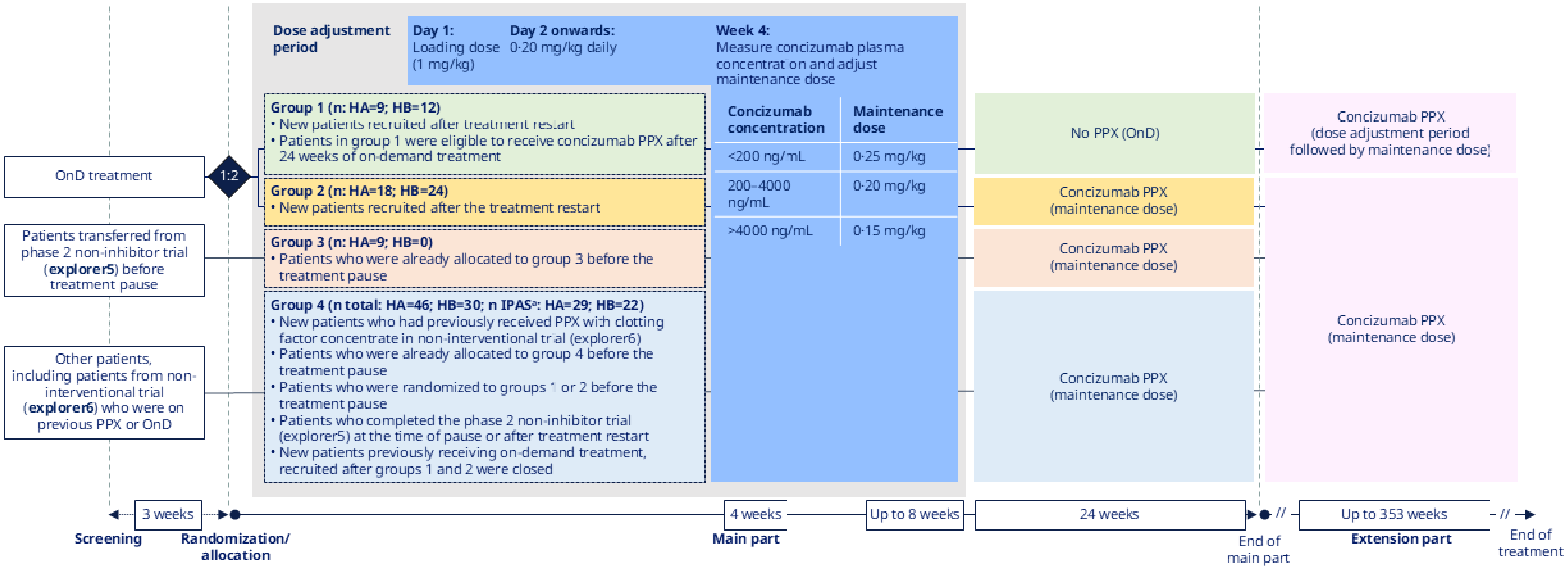

From Chowdary P et al., Concizumab prophylaxis in people with haemophilia A or haemophilia B without inhibitors (explorer8): a prospective, multicentre, open-label, randomised, phase 3a trial. Lancet Haematol 2024; 11(12): e891-e904. Please note that the extension part duration has been updated to 353 weeks at the time of writing the current article. Presented here is the explorer8 study design after the treatment restart. The extension part was followed by a 7-week follow-up period. explorer5 (NCT03196297) was a phase 2 clinical study investigating the efficacy and safety of concizumab in patients with hemophilia A without inhibitors. [a]A subset of patients in group 4 was included in the intra-patient analysis set, which included patients who were on a stable standard factor concentrate prophylaxis regimen for at least 24 weeks in the non-interventional explorer6 study (observational study; NCT03741881) and who entered concizumab treatment on the maintenance dose in explorer8. Stable prophylaxis was defined as the time after an initial period on prophylaxis treatment of at least 24 weeks. HA, hemophilia A; HB, hemophilia B; OnD, on-demand; PPX, prophylaxis.[6]

Figure S2: Summary of sport activity ratings at (A) baseline for all patients in the full analysis set, (B) baseline for patients with physical activity ratings data at the 32-week cut-off, and (C) corresponding data at the 32-week cut-off in the explorer8 study

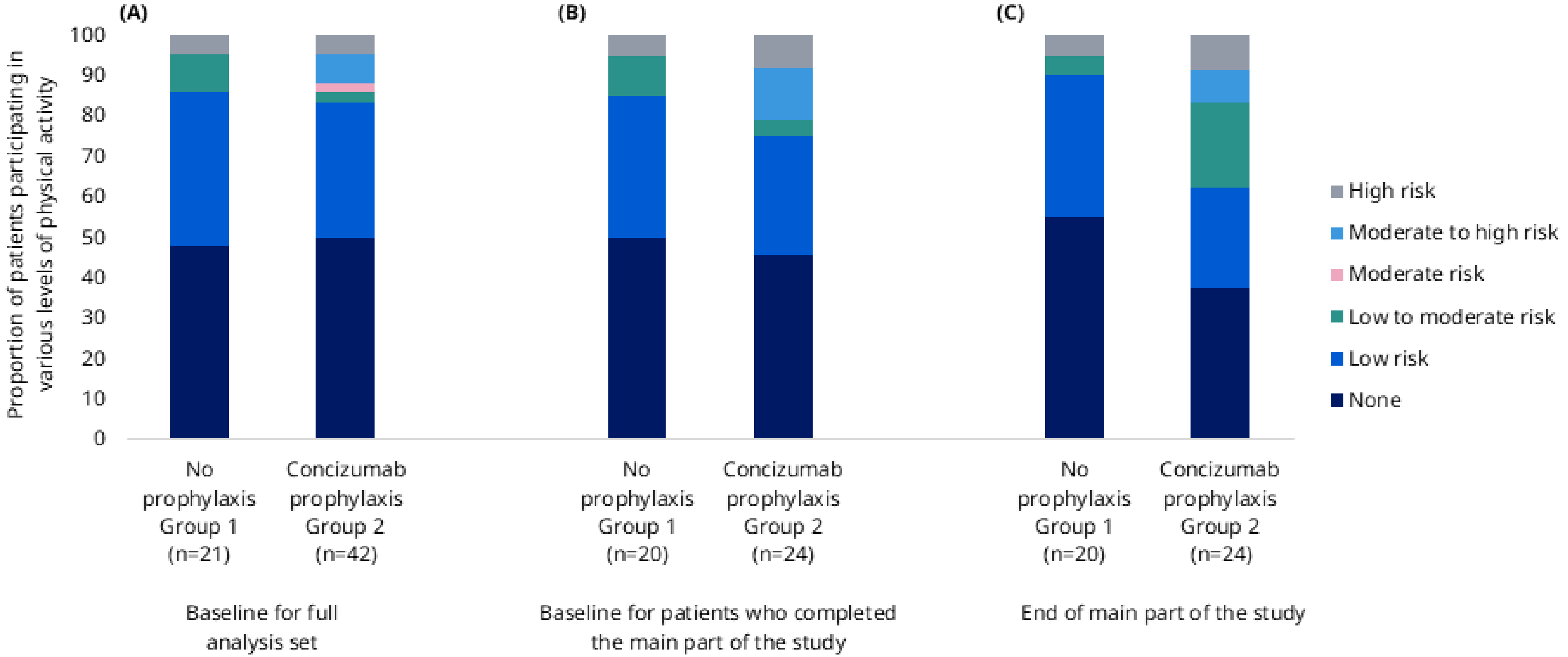

Data on sport activity ratings were collected via interviews by site staff to determine the patient's participation in low- (e.g., walking), moderate- (e.g., running/jogging) or high-risk (e.g., rugby) activities. (A) The baseline for patients who were included in the full analysis set, which includes data that were collected after treatment restart following pause due to thromboembolic events in patients in the study; this reflects the observation period in which patients are exposed to either on-demand treatment (no prophylaxis; group 1) or the new concizumab dosing regimen (concizumab prophylaxis; group 2). (B) The baseline for patients with physical activity ratings data at the 32-week cut-off of the explorer8 study. (C) Corresponding data at the 32-week cut-off of the study. Observation time during the initial concizumab dosing regimen and data collected after permanent treatment discontinuation are not included. It should be noted that the physical activity data collected during the explorer8 study overlapped with the COVID-19 pandemic, and the resulting lockdowns may have influenced patients' ability to participate in various sport activities.[5]

References

1. Staudenmayer J, He S, Hickey A, Sasaki J, Freedson P. Methods to estimate aspects of physical activity and sedentary behavior from high-frequency wrist accelerometer measurements. *J Appl Physiol (1985)* 2015; **119**(4): 396-403.
2. Castaman G, Abraham A, Angchaisuksiri P, et al. The Effect of Concizumab Prophylaxis on Target Joints, Resolution and Joint Bleeds in Patients With Hemophilia A or B With or Without Inhibitors in Phase 3 Clinical Trials. *Blood* 2023; **142**(Supplement 1): 284-6.
3. Wheeler AP, Apte S, Benson G, et al. OC 59.2 Annualized bleeding rates in hemophilia A/B and target joints: Concizumab explorer8 study. *Research and Practice in Thrombosis and Haemostasis* 2025; **9**: 102925.
4. Mahlangu J, Castaman G, Abraham A, et al. Management of breakthrough bleeding episodes in the phase 3 concizumab studies. *Research and Practice in Thrombosis and Haemostasis* 2024; **8**: 86–7.
5. Jiménez-Yuste V, Chan AKC, Obeid J, et al. Active lifestyle in patients with haemophilia A or B with and without inhibitors on once-daily subcutaneous concizumab prophylaxis: Results from the phase 3 explorer7 and explorer8 trials. *Haemophilia* 2024; **30**(S2): 3-223.
6. Chowdary P, Angchaisuksiri P, Apte S, et al. Concizumab prophylaxis in people with haemophilia A or haemophilia B without inhibitors (explorer8): a prospective, multicentre, open-label, randomised, phase 3a trial. *Lancet Haematol* 2024; **11**(12): e891-e904.
